# P-2044. Analysis of Post COVID-19 Condition After Ensitrelvir Treatment in Asymptomatic or Mild-Symptoms Only Patients in the SCORPIO Phase2b/3 Study

**DOI:** 10.1093/ofid/ofae631.2200

**Published:** 2025-01-29

**Authors:** Takumi Imamura, Hiroshi Yotsuyanagi, Norio Ohmagari, Yohei Doi, Yohei Doi, Masaya Yamato, Hiroki Sakaguchi, Hideki Yamanaka, Ryosuke Imaoka, Akimasa Fukushi, Genki Ichihashi, Yuko Tsuge, Takeki Uehara, Hiroshi Mukae

**Affiliations:** Shionogi, Osaka, Osaka, Japan; The University of Tokyo, Tokyo, Tokyo, Japan; National Centre for Global Health and Medicine, Shinjuku, Tokyo, Japan; Fujita Health University, Aichi, Aichi, Japan; Fujita Health University, Aichi, Aichi, Japan; Rinku General Medical Center, Izumisano, Osaka, Japan; SHIONOGI & CO., LTD., Osaka-shi, Osaka, Japan; Shionogi & Co., Ltd., Osaka, Japan, Osaka, Osaka, Japan; Shionogi & Co., Ltd., Osaka, Japan, Osaka, Osaka, Japan; Shionogi & Co., Ltd., Osaka, Japan, Osaka, Osaka, Japan; SHIONOGI & CO., LTD., Osaka-shi, Osaka, Japan; Shionogi, Osaka, Osaka, Japan; SHIONOGI & CO., LTD., Osaka-shi, Osaka, Japan; Nagasaki University, Nagasaki, Nagasaki, Japan

## Abstract

**Background:**

Ensitrelvir is an oral SARS-CoV-2 3CL protease inhibitor developed to treat COVID-19. In the Phase3 SCORPIO-SR trial targeting mild/moderate COVID-19 patients, the proportion of patients who experienced Post COVID-19 Condition (PCC) until Day 337 was lower in ensitrelvir groups than in placebo group [A. Fukushi et al. The Ninth ESWI Influenza Conference]. Here we report the effect of ensitrelvir on PCC in less symptomatic patients enrolled in another part, Phase2b/3.

Proportion of patients with Post-COVID-19 Condition (PCC) and risk reduction versus placebo.

**Figure:**
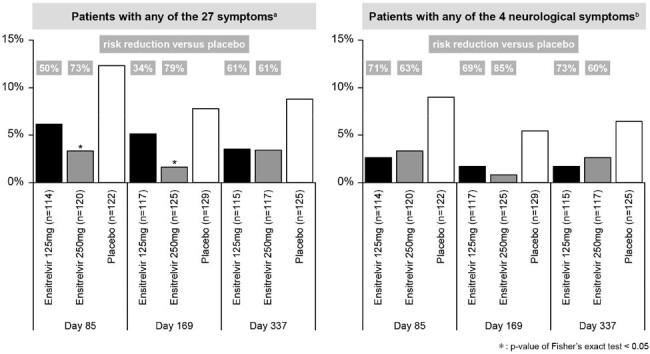

Eligible patients were randomized (1:1:1) to orally receive once-daily ensitrelvir 125 mg (375 mg on Day 1), 250 mg (750 mg on Day 1), or placebo for 5 days.

The number of patients with mild COVID-19 symptoms was 350 and the number of asymptomatic participants was 34. Among asymptomatic participants (9 in ensitrelvir 125mg, 13 in ensitrelvir 250mg, and 12 in placebo group), 2 participants had PCC (one in 125 mg and the other is placebo group).

Data are summarized for patients who completed the PCC questionnaire for at least one timepoint in the intention-to-treat population.

a Stuffy nose, runny nose, sore throat, shortness of breath, cough, low energy or tiredness, muscle or body aches, headache, chills or shivering, feeling hot or feverish, nausea, vomiting, diarrhea, loss of smell, loss of taste, muscle weakness, hair loss, palpitations, joint pain, decreased appetite, dizziness, chest pain, skin rash, difficulty with concentration and thinking, difficulty in reasoning and solving problems, memory loss, or insomnia.

b Difficulty with concentration and thinking, difficulty in reasoning and solving problems, memory loss, or insomnia.

**Methods:**

This multinational, multicenter, randomized, double-blind, placebo-controlled study was conducted in 2022 when the Omicron-variant was dominant. Individuals with confirmed SARS-CoV-2 infection, regardless of their vaccination status or risk factors for severe disease, were enrolled. This study targeted patients with diagnosis of COVID-19 asymptomatic/mild symptoms who were screened but not enrolled in Phase3 SCORPIO-SR study. Participants were randomized to either ensitrelvir 125 mg, 250 mg, or placebo. PCC was defined as a condition reported as “not returned to pre-COVID health” with at least one mild/more severe symptom among prespecified 27 symptoms on Day 85, 169, and 337. Investigators and patients remained blinded to drug assignment up to Day 337.

**Results:**

In total, 124 ,129 ,131 patients were assessed in the ensitrelvir 125 mg, 250 mg, and placebo groups, respectively (mean age, 38.8–41.6 years; men, 51.1–61.2%; 89.9–93.1% had vaccination history). Proportion of patients with PCC on Day 337 for any of the 27 symptoms was 3.5%, 3.4% and 8.8% in ensitrelvir 125 mg, 250 mg and placebo groups, respectively. Ensitrelvir 125 mg and 250 mg showed 60.5% and 61.1% risk reductions versus placebo, respectively. For any of the 4 neurological symptoms, proportion of patients with PCC on Day 337 was 1.7%, 2.6% and 6.4% in ensitrelvir 125 mg, 250 mg and placebo groups, respectively. Risk reductions versus placebo were 72.8% and 59.9% in ensitrelvir 125 mg and 250 mg, respectively (Figure).

**Conclusion:**

It is suggested that ensitrelvir treatment may reduce PCC in this limited subpopulation with asymptomatic/mild disease.

**Disclosures:**

Takumi Imamura, M.Math, Shionogi & Co., Ltd.: Stocks/Bonds (Private Company) Hiroshi Yotsuyanagi, MD PhD, Japanese Society of Infectious Disease: President|Shionogi & Co., Ltd.: Board Member|Shionogi & Co., Ltd.: Lecture fees, travel and meeting support, and Chairs in sponsored symposiums|ViiV Healthcare: Lecture fees; Chairs in sponsored symposiums Yohei Doi, MD, PhD, bioMerieux: Lecture fees|Entasis: Grant/Research Support|Fujifilm: Advisor/Consultant|Gilead Sciences: Advisor/Consultant|GSK: Advisor/Consultant|KANTO CHEMICAL CO.,INC.: Grant/Research Support|KANTO CHEMICAL CO.,INC.: Patent for genotyping kit|MeijiSeika Pharma: Advisor/Consultant|Moderna: Advisor/Consultant|MSD: Lecture fees|Pfizer: Advisor/Consultant|Shionogi & Co., Ltd.: Grant/Research Support|Shionogi & Co., Ltd.: Lecture fees Masaya Yamato, MD, PhD, Shionogi & Co., Ltd.: Lecture fees Hiroki Sakaguchi, MA, Shionogi & Co., Ltd.: Stocks/Bonds (Private Company) Hideki Yamanaka, n/a, Shionogi & Co., Ltd.: Stocks/Bonds (Private Company) Ryosuke Imaoka, n/a, Shionogi & Co., Ltd.: Stocks/Bonds (Private Company) Akimasa Fukushi, n/a, Shionogi & Co., Ltd.: Stocks/Bonds (Private Company) Genki Ichihashi, MA, Shionogi & Co., Ltd.: Stocks/Bonds (Private Company) Yuko Tsuge, MSc, Shionogi & Co., Ltd.: Stocks/Bonds (Private Company) Takeki Uehara, Ph.D., Shionogi & Co., Ltd.: Stocks/Bonds (Private Company) Hiroshi Mukae, M.D., Ph.D., AstraZeneca: Lecture fees|Gilead Sciences: Lecture fees|GSK: Lecture fees|MSD: Advisor/Consultant|MSD: Lecture fees|Pfizer: Lecture fees|Shionogi & Co., Ltd.: Advisor/Consultant|Shionogi & Co., Ltd.: Grant/Research Support|Shionogi & Co., Ltd.: Lecture fees

